# Mood, Activity Participation, and Leisure Engagement Satisfaction (MAPLES): a randomised controlled pilot feasibility trial for low mood in acquired brain injury

**DOI:** 10.1186/s40814-020-00660-8

**Published:** 2020-09-22

**Authors:** Andrea Kusec, Fionnuala C. Murphy, Polly V. Peers, Cara Lawrence, Emma Cameron, Claire Morton, Andrew Bateman, Peter Watson, Tom Manly

**Affiliations:** 1 MRC Cognition and Brain Sciences Unit, University of Cambridge, 15 Chaucer Road, Cambridge, CB2 7EF UK; 2 Evelyn Community Head Injury Services, Cambridgeshire Community Services, Dynamic Health Building, Brookfields Hospital, 351 Mill Road, Cambridge, CB1 3DF UK; 3 The National Hospital for Neurology and Neurosurgery, University College London Hospitals NHS Trust, Post Box 113, Queen Square, London, WC1N 3BG UK; 4 School of Health and Social Care, University of Essex, Wivenhoe Park, Colchester, CO4 3SQ UK

**Keywords:** Acquired brain injury, Traumatic brain injury, Depression, Executive function, Neurorehabilitation

## Abstract

**Background:**

Acquired brain injury (ABI) affects approximately 79.3 million individuals annually and is linked with elevated rates of depression and low mood. Existing methods for treating depression in ABI have shown mixed efficacy. Behavioural activation (BA) is a potentially promising intervention. Its premise is that individuals with low mood avoid planning and engaging in activities due to low expectations of a positive outcome. Consequently, their exposure to positive reinforcement is reduced, exacerbating low mood. BA aims to break this cycle by encouraging activity planning and engagement. It is unknown whether cognitive demands of traditional BA may undermine efficacy in ABI. Here, we assess the feasibility and acceptability of two groups designed to increase activity engagement. In the activity planning group (traditional BA), the importance of meaningful and positive activity will be discussed and participants encouraged to plan/engage in activities in everyday life. The activity engagement group (experiential BA) instead focuses on engagement in positive experiences (crafts, games, discussion) *within* the group. The primary aims are to evaluate the feasibility and acceptability of the two groups in ABI. A secondary aim is to explore relative efficacy of the groups compared to an equivalent period of waitlist controls.

**Method:**

This study outlines a parallel-arm pilot feasibility trial for individuals with low mood and ABI that compares a traditional vs experiential BA group vs waitlist controls. Adults (≥ 18 years) will be recruited from local ABI services and randomised to condition. Feasibility and acceptability will be assessed via recruitment, retention, attendance and participant feedback. Groups will be compared (pre- and post-intervention and 1 month follow-up) by assessing self-reported activity engagement. Secondary outcomes include self-report measures of depression, anxiety, post-traumatic distress related to the ABI, motivation, participation and sense of control over one’s life.

**Ethics and dissemination:**

The trial has been approved by the Health Research Authority of the NHS in the UK (East of England—Cambridge Central, REF 18/EE/0305). Results will inform future research on interventions for mood in ABI and be disseminated broadly via peer-reviewed journals, conference presentations and social media.

**Trial registration:**

ClinicalTrials.gov, NCT03874650 pre-results. Protocol version 2.1, March 5, 2019

## Background

Acquired brain injury (ABI) refers to damage to the brain from a blow to the head (traumatic brain injury; TBI), from an interruption to the brain’s blood supply (stroke) or oxygen supply (anoxia), or as a result of pressure from a growing tumour [1]. In the United Kingdom (UK), TBI’s affect 531 per 100,000 individuals per year [2] and stroke approximately 115 per 100,000 individuals [3]. Anoxic brain damage due to cardiac arrest affects roughly 50 people per 100,000 annually [4], brain tumours a further 7 per 100,000 [5] and encephalitis approximately 5 per 100,000 per year [6]. The cost of just TBI and stroke in the UK is £15 billion and £8.9 billion per year, respectively [7, 8]. Taken together, ABI is a leading cause of long-term disability worldwide [9] and presents a considerable public health challenge.

ABIs can have far-reaching negative effects on an individual’s physical, cognitive, behavioural, emotional and social status [2, 10]. The purpose of rehabilitation is to enable those with an ABI to successfully reintegrate into the community by developing essential skills necessary for a patient’s goals [11]. However, positive long-term outcome of rehabilitation can be significantly reduced by depression [12, 13]. In the UK, approximately half of all people with an ABI—354 of the estimated 708 individuals per 100,000—will experience depression in the first year after their injury, of those with ABI with depression, roughly 66% do not fully recover from their depressive symptoms [14]. Individuals with an ABI and depression are more likely to experience greater difficulties in many aspects of day-to-day function [8, 9], including poorer quality of life, impaired overall cognitive function, reduced physical activity and engagement in activities of daily living and a higher mortality rate [15–18]. Alarmingly, individuals with an ABI are at least 3 times as likely to die by suicide relative to the general population [19].

Clearly, there is an urgent need to develop effective interventions for depression in ABI populations. Though existing therapies for depression such as cognitive behavioural therapy (CBT) have a strong evidence base in the general population, they place heavy demands on skills often compromised in ABI, such as comprehension, memory and mental flexibility, which may help account for the mixed outcomes for CBT in ABI [20]. A promising alternative with lower demands and established efficacy in the non-ABI population is behavioural activation (BA). Individuals with depression have difficulties anticipating and imagining positive future activities and tend not to plan or engage in them [21–23] for reasons that include reduced motivation, fatigue, or fear of negative consequences. This limits their exposure to opportunities for positive reinforcement which can exacerbate low mood [24]. In BA, rather than waiting until their mood improves spontaneously, individuals are encouraged to plan and engage in meaningful and valued activities and overcome barriers to their occurrence. Despite its simplicity, BA’s effectiveness in the non-ABI population is on par with medication and CBT, with a pooled effect size of 0.78 between BA and control conditions [25–27]. Since BA has relatively low cognitive demands compared with, say, CBT, it may be particularly well suited to individuals with ABI. Furthermore, a core component of BA is problem-solving barriers related to activity engagement, which is well suited to addressing the considerable cognitive, physical, motivational and societal challenges that are common in ABI. Reduced activity engagement is an important factor contributing to the elevated rates of depression and low mood in ABI [28–32] and thus interventions aimed at improving activity engagement are well suited toward this population. Promising work examining BA in the specific context of stroke is already underway [33, 34]. In a re-analysis of randomised controlled trial data, Bombardier et al. [35] concluded that environmental rewards from daily activities correlated with decreased depression, suggesting the BA-style interventions may be appropriate in TBI as well. This is the first trial of which we are aware to explore BA with respect to the wider ABI population and to do so using group rather than individual approaches.

An important issue given the mixed efficacy of other psychological interventions for mood in ABI is whether the cognitive demands of BA, although notably lower than other approaches, may still undermine its effectiveness. For example, BA requires participants to consider the relationship between activity engagement and mood. Participants are asked to identify and schedule meaningful activities, remember to complete these activities and then reflect on successes, failures and barriers. In BA, it is hoped individuals acquire the skills and motivation to maintain activity engagement after the course of BA sessions have concluded. There is a risk, therefore, that despite the many features included to facilitate engagement, individuals with marked memory and/or organisational difficulties arising from ABI may struggle to complete the exercises and experience feelings of failure and inadequacy. For this reason here we will compare a traditional BA group (activity planning group) with a more “hands on” experiential approach (activity engagement group). In the latter group, participants will be engaging in positive activities *within the group* (crafts, games, art, discussions) without placing any demands on planning, engaging and reporting back on activities from everyday life. For these reasons, the activity engagement group may be better accepted than the activity planning group. Alternatively, the activity planning group may be better accepted if it is perceived as “proper therapy” directed at long-term change, rather than the temporary engagement offered in the activity engagement group. However, the activity engagement group is similar to many social or activity-based groups already offered by local charities including to people with ABI. As has been pointed out [36], such groups are rarely if ever evaluated relative to clinical services. Exploring differences in feasibility and research outcomes between the two types of groups are therefore key aims of this trial.

BA has shown strong effects both individually (*d* = 0.78) and in group (*d* = 0.74) settings in the non-ABI population [25, 37, 38] as well as preliminary support (*d* = 0.27 to 0.71) in one-to-one therapy in stroke [34], but this is the first group BA evaluation, of which we are aware, in ABI. There are clear economic advantages to groups compared with individual therapy [39]. Beyond this, treatment waitlists are reduced by utilising group formats, hence potentially reducing long-term costs such as days of missed work [40–42]. In addition, when they work well, the supportive dynamics of groups of people with similar challenges can enhance therapeutic effects [36, 40].

Therefore, the proposed trial will investigate the feasibility of two activity-based interventions and whether explicitly training individuals with an ABI to plan and engage in meaningful activities is superior in terms of acceptability and practicality, and explore whether either intervention affects activity level and mood compared with an equivalent period of waitlist controls.

### Objectives

The primary objective of the Mood, Activity Participation, and Leisure Engagement Satisfaction (MAPLES) trial is to determine the feasibility and acceptability of two activity-based group interventions in individuals with an acquired brain injury (ABI) with low mood. This will be based on participant retention from baseline to 1-month post-intervention, acceptability of the proposed skills in the group sessions and assessments, and participant feedback from the exit interview.

The secondary objective of the MAPLES trial is to explore whether *either* traditional or experiential behavioural activation potentially leads to changes in mean activity level and related outcomes including depression, anxiety, post-traumatic stress, motivation, participation and sense of control, compared to a waitlist control condition at the group level in individuals with an ABI.

### Trial design

The MAPLES trial is a parallel-arm randomised controlled trial with nested qualitative research. Participants will be allocated on a 1:1:1 ratio.

## Methods

### Participants, interventions and Outcomes

#### Study setting

Approximately 60 individuals with an ABI will be recruited within the Cambridgeshire region of East of England, UK; specifically those who are clients of Cambridgeshire Community Services (CCS) NHS Trust. The trial will take place in community-based clinics within CCS. The list of trial sites can be obtained as part of the trial registration documentation (NCT03874650) on clinicaltrials.gov.

#### Eligibility criteria

Participants will be included in the trial if they meet the following:

1. Have a diagnosis of an ABI^1^
2. Are a client of Cambridgeshire Community Services
3. Are 18 years of age or older
4. Speak and comprehend English
5. Are a minimum of 3 months post-ABI
6. Are identified as having low mood/reduced activity level. These will be identified by the following:
  - A score of at least 7 on the depression subscale of the Hospital Anxiety and Depression Scale (HADS-D [43])
  - Clinician report (i.e. through the clinician’s own administration of the HADS-D within the past 3 months of screening date or clinical interview that has indicated a client has low mood/would benefit emotionally from increased activity level).

Participants will be excluded from the trial if they:

1. Are incapable of attending to and/or understanding the intervention materials, based on clinical judgement from referrer (i.e. has receptive aphasia, does not have capacity to consent and are unable to live independently)
2. Have a diagnosis of dementia or other neurodegenerative disorder
3. Are currently undergoing or due to undergo a psychological intervention for low mood or depression (e.g. CBT) during the timeframe of the trial
4. Unstable psychotropic medication (i.e. has started or changed medications within the past 6 weeks)
5. Are actively suicidal (i.e. have attempted suicide in the past 3 months, currently self-harm and/or have suicidal intentions in the near future), as identified by the referring clinician

#### Interventions

The MAPLES trial consists of two interventions, each designed to facilitate engagement in pleasant and meaningful activities through two different approaches—the activity planning group and the activity engagement group. These groups will each meet once weekly for 1 to 1.5 h over 8 weeks. Group sizes will be at minimum 3 individuals and maximum 6 individuals. In order to ensure that any benefits from each group are beyond treatment as usual, the MAPLES trial will also run a waitlist control group, whereby participants will receive standard care for 8 weeks and then be re-randomised to either the activity planning or activity engagement group (see the “Allocation—sequence generation” section for details on allocation).

#### Therapist training and characteristics

Both the activity planning group and the activity engagement group will be facilitated by AK. AK has an undergraduate degree in psychology and a Master’s degree in rehabilitation science that involved conducting research with individuals with an ABI and has 8 years’ experience volunteering with individuals with an ABI. AK has received training on the intervention (approximately 6 h) and regular supervisions from a registered clinical psychologist (TM) and from senior members of the research team (FCM and PVP).

#### Activity planning group

The activity planning group will take a “traditional” BA approach to increasing engagement in meaningful and positive activities. Generally, those receiving BA training develop and maintain a schedule based on activities that have been enjoyable, pleasant, meaningful, or interesting in the past [25]. Clients are instructed to monitor their daily mood and participation in these activities to identify the connection between them and taught how to increase the frequency and quality of positive events [44, 45]. Along with the above, participants assigned to the activity planning group will also learn about identifying and challenging counter-productive patterns of avoidance and procrastination as well as consider barriers that may be a particular problem for people with ABI, such as distraction and goal neglect, alongside strategies that may help to overcome these [46, 47]. The activity planning group will consist of weekly 1- to 1.5-h group sessions over 8 weeks, covering 8 overarching themes:

1. Introduction to group therapy
  Participants are given an overview of behavioural activation and the relationship of activity level to mood. Participants are also provided information on why planning activities is difficult after brain injury and are introduced to mood monitoring and monitoring lapses in attention.
2. Identifying enjoyable activities
  Participants discuss their monitoring results and learn about the relationship of mood to attention. Participants begin identifying what goals they have for themselves, activities that align with their goals and values and schedule in the first activity using a step-by-step approach.
3. Changing habits and planning pleasurable activities
  Participants problem solve their activity from the past week and identify barriers and facilitators related to activity completion and discuss how the activities made them feel. Participants then learn about how activities become habitual and what personal triggers prevent completion of an activity. Participants plan in another activity.
4. Goal review and balancing enjoyable and routine activities
  Participants review their activity from the past week and learn how to prevent barriers to activity completion. Participants then review their goals from week 2 and their daily schedules to reflect on whether there are activities that are making them feel down that could be altered or removed. Participants plan in another activity.
5. Identifying solutions to goal attainment
  Participants review their activity from the past week. Participants learn to identify avoidance patterns and how to create solutions to personal triggers and avoidance patterns. Participants learn how to prevent distraction from a task and plan in another activity.
6. Increasing mastery and managing fatigue
  Participants review their activity from the past week. Participants discuss the potential benefits of consistently challenging themselves to increase activity level and the risks of not increasing mastery. Participants learn to recognise warning signs of fatigue when attempting to increase activity level. Participants plan in another activity and are encouraged to increase overall activity level.
7. Active approaches to engagement
  Participants review their activity from the past week. Participants discuss the importance of social relationships in maintaining a good mood and barriers to taking initiative to plan activities with other people. Participants practice directly initiating activities with others and are encouraged to plan in an activity with other people.
8. Relapse prevention
  Participants review their activity from the past week. Participants then review the content from the past 7 weeks and list individual “take home” messages from the group. Participants identify personal triggers for relapse and create strategies to overcome triggers should they encounter them. Participants are encouraged to keep up activity scheduling and planning in a stepwise fashion.

The above modules were designed in line with typical BA interventions (for a review, see Kanter et al. [24]) as well as accepted and effective rehabilitation strategies for executive function impairment [47, 48]. Individuals with an ABI and their family members were also interviewed to determine what barriers and facilitators are present when engaging in day-to-day and meaningful activities.

#### Activity engagement group

The activity engagement group will take a “hands on” experiential approach to increasing activity level. Individuals randomised to this arm will meet weekly for 1 to 1.5 h for 8 weeks and engage in various potentially rewarding or meaningful social activities such as board games and crafts. Participants in this group will not receive specific training on increasing activity level outside of these weekly sessions or overcoming barriers to participation. Instead, the aim is that participants experience positive reinforcement within the activity engagement group without explicitly scheduling activities outside of the group.

At the beginning of the 8 weeks, participants are told of the MAPLES trial purpose and that one approach to increasing activity level is to “learn by doing” and that as part of the group they will take part in potentially interesting/creative activities. Participants are offered suggestions by the group facilitator (AK), such as board games, t-shirt making, puzzles, painting, “pub quizzes”, figurine painting, origami/papercraft, and clay sculpting. The activities in this group were selected based on typical activities organised by local ABI charities to promote engagement in meaningful activities. Participants are also told they are welcome to suggest activities to complete within the group (e.g. social discussion only, customising photo frames). There are no restrictions for activities to only be conducted within one session—for example, participants are free to choose to play board games throughout the 8 weeks. We view this as analogous to traditional BA whereby participants choose what activities they wish to plan outside of the group.

The activities within the group are meant to be activities that are feasible to recreate and complete within a health care facility or community-based charity group and thus should not have excessive financial costs or limitations due to the structure of the facilities and would not risk exclusion of participants who might be physically incapable of completing the activities. For example, completing paintings would be possible within a group setting, but organising cooking sessions, trips outside the health care facility and expensive activities that would require a large initial investment and sufficient dexterity (e.g. carpentry) would not be suitable. Given the focus of activity completion within the group, this may implicitly or explicitly challenge negative beliefs about engaging in meaningful activities and thus participants may maintain increased activity level and enhanced mood past the end of the group.

#### Waitlist control group

With respect to our secondary aims, in order to separate any benefits gained from the activity planning or activity engagement groups from benefits that might occur spontaneously over an 8-week period, approximately a third of participants will be first assigned to the waitlist group. Given that individuals with an ABI might have varying aspects/types of clinical care, the decision was made not to ask participants to discontinue contact with their normal clinical services. Hence, the waitlist group forms a “treatment as usual” control arm. The waitlist group will run for 8 weeks, after which participants will complete a second baseline assessment and will then immediately be re-randomised into either the activity planning or activity engagement group.

#### Intervention criteria

The above named interventions will be discontinued in the event that participation in the groups results in harm or deterioration of participants (e.g. worsening depression). We will monitor participants in two ways. Firstly, where there are instances of reported thoughts about or incidents of self-harm in the group, these will be recorded and managed through clinical supervision (e.g. to inform whether relevant clinical services are informed). Secondly, changes in mood questionnaire scores will be examined between baseline, time 2 and time 3 to establish whether the above criteria for discontinuing the study have been reached. Where discontinuing criteria have not been reached but individual participants show deterioration leading to concern, this will be managed on a case-by-case basis through clinical supervision, discussion and decisions about whether continuing to participate is in that individual’s best interests and whether clinical services need to become engaged. Adherence to the treatment manual and trial protocol is managed by the steering committee and is determined by audio recordings of group sessions. Although the core elements as listed above for each group will not change outside of risk of harm to the participant, minor changes may be introduced as the sessions progress. Examples could include changes to the text (homework sheets), images (photos used to illustrate topic) and activities (games within the activity engagement group) in response to in-session acceptability of materials. Changes to worksheets will be tracked. Participants will be allowed to seek treatment throughout the course of the intervention for medical difficulties (e.g. physiotherapy appointments), and although we do not actively recruit individuals receiving psychological services, if a participant already enrolled in the trial decides to refer themselves to psychological services, we would not discourage this. We will emphasise that attendance within the MAPLES trial is still possible if the participant has queries about attending both study sessions and other clinical services. We would encourage the participant to discuss trial participation with their practitioner and remind the participant they are free to withdraw from the study at any stage. Any new involvement in psychological services will be documented and reported as part of the trial data. This is viewed to be the most ethical position whilst still maintaining sufficient documentation of other services accessed within the trial.

#### Treatment integrity and fidelity

Compliance with the trial protocol will be managed primarily by regular review of recruitment strategies, intervention delivery, and assessment administration. These activities will be overseen by a Steering Committee which has been assembled for this purpose and will meet regularly over the course of the trial. The meetings will include recruitment site initiation sessions to ensure that all individuals involved in recruitment are identifying participants in a consistent manner. Regular review of documentation of evidence of eligibility will be conducted by a member of the research team not involved in the screening and recruitment process nor conducting the intervention.

All group sessions will be audio recorded in order to code the sessions for intervention fidelity. A member of the research team not conducting the intervention will listen to audio recordings of each session in order to determine adherence to the protocol. Fidelity in the activity planning group will be assessed by (1) identifying whether the therapist covered the components outlined in the fidelity checklists (see Additional files 1 and 2) in a suitable manner (i.e. not skimmed) and (2) how consistently techniques were applied across group cohorts. Given the inherently flexible nature of the activity engagement group, general principles rather than specific content will be used to evaluate fidelity. For the activity engagement group, the content covered in the activity planning group should *not* be mentioned, and the core components as listed in Table 1 should be present in each session.

**Table 1:** Core components of each group

Activity planning group	Activity engagement group	Waitlist control group
• Activity scheduling and monitoring	• Participant autonomy	• No contact with research team during wait
• Identifying avoidance patterns	• Direct experience of selected activity	• No in-depth information about either study group
• Problem-solving barriers to activation	• Focus on social engagement	• Not actively discouraged from continuing other forms of care
• Step-by-step activity planning	• Non-linear group structure	
• Enhancing planning through goal management	• No explicit planning of activities	

Assessment compliance at each time point (i.e. completing the full outcome measure battery at baseline, post-intervention, and 1-month post-intervention) will be completed using a checklist and comment section to input any information as to why an assessment was not fully completed. Any protocol deviations, whether accidental or intentional, will be documented and reported to the principal investigator (TM), and major concerns to both TM and sponsor immediately.

#### Outcomes

The primary objective of this trial is to examine feasibility and acceptability of the activity planning group and the activity engagement group to inform the design of subsequent larger-scale studies. The secondary objective of this trial is to explore the potential therapeutic benefit of the activity planning group and the activity engagement group relative to the waitlist group.

##### Primary objective—primary outcome measure

There is no single primary outcome measure to determine feasibility and acceptability of the MAPLES trial. These will be determined based on (1) ability to meet recruitment targets, (2) attrition levels across the three groups, (3) group attendance, (4) a post-study questionnaire whereby participants are asked to rate aspects of their group (i.e. perceived benefits of each session, suggestions for improvements), and (5) qualitative data from the exit interview from a subset of participants, which includes questions about participants’ experience within each group.

These outcomes will be evaluated based on specific targets for each objective, as below:

1. For each cohort of groups, a minimum of 9 people and maximum of 18 participants recruited
2. Attrition levels less than 20% across the three arms of the trial
3. Participants attending at least 5 of 8 sessions within the activity planning and activity engagement group
4. Positive ratings of group aspects in the post-study questionnaire
5. Minimal barriers to attendance and engagement in groups, as evident in the qualitative data

##### Secondary objective—research outcome measure

To determine the secondary objective, the potential therapeutic benefit of the activity planning and activity engagement groups, the *Behavioural Activation for Depression Scale* (BADS [49]) will be used. Additional descriptives of secondary outcome measures between groups will also be conducted. Please see the “Methods: data collection, management, and analysis” section for a description of the BADS and the secondary outcome measures.

#### Participant timeline

**Fig. 1:**
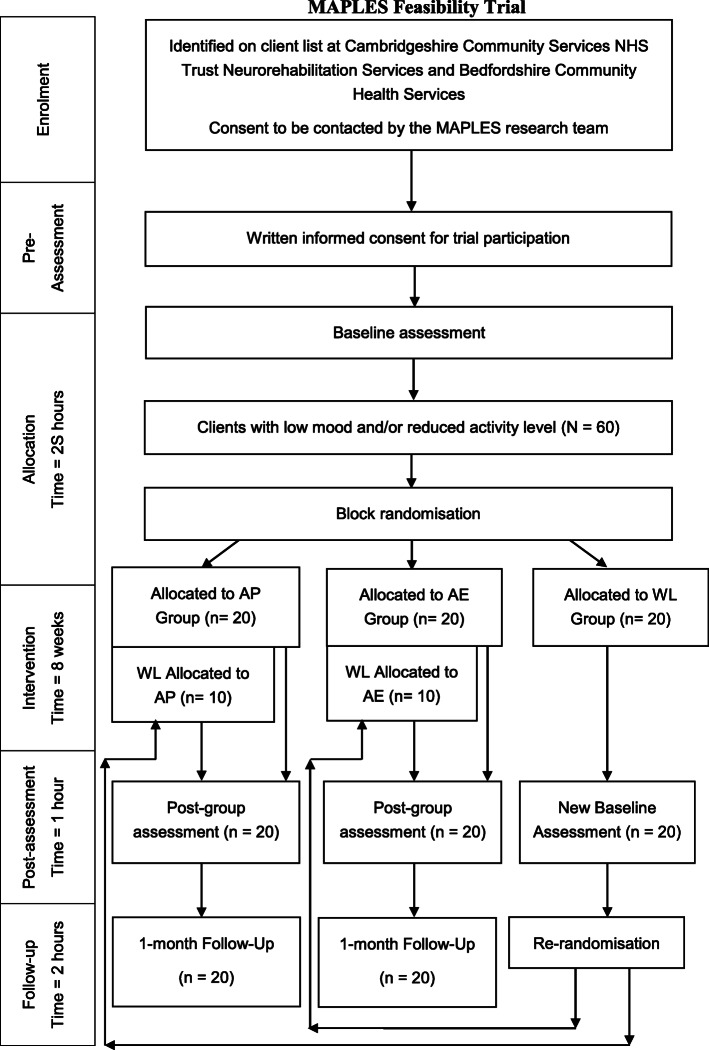
Participant flow diagram with expected numbers. AP, activity planning; AE, activity engagement; WL, waitlist control

Participants will be recruited for approximately 18 months, and enrolled on a rolling basis. No run-ins or washouts are planned as part of this trial. After the research team has received a referral, participants will be scheduled for a baseline assessment within 1 month maximum of the current phase of groups. Following the end the first phase of the activity planning group, activity engagement group, and waitlist control group, time 2 and time 3 assessments will be held concurrently with new time 1 baseline assessments for the subsequent phases, which will lead to an approximately 1-month gap in between phases of groups. Please see Fig. 1 for an overview of the participant timeline. In effect, only one group of both the activity planning and activity engagement group will be running at any given time, with an aim to enrol 6 cohorts of each group during the recruitment timeframe.

#### Sample size

Although typically power calculations are conducted for trials, the primary objective of the MAPLES trial is to determine the feasibility and acceptability of the intervention. Based on our previous experience [50, 51] approximately 20 participants per arm (a total of 60 participants) would provide a sufficient balance between determining feasibility and exploring potential mediators of outcome between the activity planning group and activity engagement group that can guide the development of a future larger-scale trial.

#### Recruitment

All participants will be recruited through ABI clinical services within Cambridgeshire Community Services (CCS) NHS Trust. The clinical staff within CCS will first identify participants from their records of clients currently or previously associated with the service. Upon identification of potentially suitable clients, the clinical staff will either (1) provide an invitation letter in person to a client that meets eligibility criteria or (2) post or email invitation letters to any client meeting eligibility criteria. The invitation letter will provide a brief overview of the trial and its procedures and a summary of the two groups. If clients are interested they will be given the option to give written consent for their clinician to pass on client contact information to the research team who would then approach the client (via phone call, SMS, email, mail, or in person meeting).

Once contact is made, the research team will first confirm eligibility criteria, after which an appointment will then be made to take written informed consent and conduct a baseline assessment using the measures specified below.

### Method—assignment of interventions

#### Allocation—sequence generation

The MAPLES trial will use pre-determined blocked randomisation. The trial statistician will conduct a computer-generated, pseudo-random allocation prior to beginning recruitment. Each participant will be assigned a code, whereby A = activity planning group, B = activity engagement group and WL = waitlist control group. Participant blocks are of varying length, unknown to all authors and researchers with the exception of the trial statistician (PW). Varying block lengths were used in order to reduce the likelihood of researchers guessing the group allocation of participants based on the allocation of previous participants.

Participants first assigned to the waitlist control group will be re-randomised to either the activity planning group or the activity engagement group again using pre-determined blocked randomisation by the trial statistician prior to commencement of the trial.

#### Allocation concealment mechanism and implementation

Following sequence generation, the trial statistician will simply write each code onto an individual card and seal it into an opaque envelope marked only with the participant number. Once generated, these envelopes will then be passed onto the researcher conducting the baseline assessments and participant enrolment. After conducting the baseline assessment, the researcher will simply open the next envelope in sequence. A second batch of 20 opaque sealed envelopes that indicate whether the participant is to be re-allocated to the activity planning group (A) or the activity engagement group (B) will be created. This randomisation is conducted independently of the first randomisation procedure. Again, once a second baseline has been performed by the researcher (required prior to re-randomisation), the researcher will open the second envelope in sequence to determine allocation for those first assigned to the waitlist control group. As all randomisation envelopes are created prior to enrolment, we assume a priori that those allocated to the waitlist control group will experience an improvement once re-randomised to either the activity planning group or activity engagement group and completing either group, hence assuming they require further treatment. In order to mitigate bias, all researchers conducting outcome assessments will not be involved in opening any of the envelopes for participants.

#### Blinding

Clinicians recruiting participants will be unaware of what group participants will be assigned to. In this sense, randomisation is conducted blind to any information about the participants and cannot influence or be influenced by the outcome of the baseline assessments. Given that it is a psychological intervention, it will be impossible to blind either the researcher delivering the intervention or the participant to what condition they have been assigned to.

The researcher conducting the outcome assessments (time 2 and time 3) will be blinded to the group that each participant has attended. If unblinding occurs prior to the time 2 and time 3 assessments (e.g. participant forgets to not inform researcher, gives information that makes it obvious what group they were in), then another researcher will be assigned to conduct the time 2 and time 3 assessments, provided a feasible timeframe and availability of researchers. If unblinding occurs *within* one of the time 2 and time 3 assessments (e.g. participant mentions group status whilst the researcher is helping the participant complete the outcome measures), then such an incident will be recorded and reported in the final paper.

As the exit interview will ask specifically for feedback on their experience within either the activity planning or activity engagement group, it will be impossible to blind the researcher or the participant as to what condition they have been assigned to. Therefore, the exit interview will be conducted only after the time 3 outcome measures have been completed.

### Methods: data collection, management and analysis

#### Data collection methods

The outcome measures battery will be administered at baseline (time 1), post-intervention (time 2) and 1-month post-intervention (time 3) either in person, via post, or using online questionnaire software as per the convenience of the participant. Participants who are first randomised to the waitlist control group will complete a second baseline assessment prior to being re-randomised into either the activity planning group or the activity engagement group. All researchers conducting assessments will receive training relative to working with individuals with an ABI, and a walk-through of the assessment protocol.

Demographic information, including recruitment site, age, gender, years of education, ethnicity and the presence of a caregiver at home, is also recorded. At the time of written informed consent, participants will also be asked for consent to access their CCS medical records for details of the nature of their injury such as injury type and location within the brain. Participants who refuse to give access to their medical records will still be welcome to participate in the trial. The data collected from CCS medical records includes the following:

1. Date and type of ABI (e.g. TBI, stroke, hypoxia) and mechanism/cause (e.g. road traffic accident, high blood pressure, heart attack)
2. Any details on parts of the brain affected and how indicated (e.g. frontotemporal damage, CT scan)
3. Any details on length of stay in acute care
4. Date of initial assessment within CCS and rehabilitation services received
5. Severity of injury and how indicated (e.g. Glasgow Coma Scale scores, Loss of Consciousness, Post-traumatic Amnesia, Stroke Severity Scale)
6. Any health-related comorbidities (e.g. diabetes, alcohol dependence)
7. Any available data on the Dysexecutive Questionnaire [52, 53] and the European Brain Injury Questionnaire [54], both self and informant
8. Any available data on neuropsychological assessment (e.g. Wechsler Adult Intelligence Scale)
9. Any information on previous diagnosis of depression or other mental health disorder and treatment of depression (e.g. counselling, psychologist, therapist)
10. Any information on antidepressant medication (dosage and duration)

#### Secondary objective—research outcome measures

The *Behavioural Activation for Depression Scale* (BADS [49, 55];) is a 25-item measure of avoidance and activation behaviours considered to underlie depression. Participants are asked to give responses based on the previous week. The BADS consist of 4 subscales: activation (“I was an active person and accomplished the goals I set out to do”), avoidance/rumination (“I did things to avoid feeling sadness or other painful emotions”), work/school impairment (“My work/schoolwork/chores/responsibilities suffered because I was not as active as I needed to be”) and social impairment (“I was not social, even though I had opportunities to be”). Items are rated on a 7-point Likert scale, ranging from 0 (not at all) to 6 (completely). Total scores can either be calculated per subscale, or the scale total can be used. Higher scores indicate greater behavioural activation. Total scores can range from 0 to 150. The BADS has good factor structure, internal consistency and test-retest reliability as well as good construct validity [49, 55].

#### Secondary outcome measures

The *Hospital Anxiety and Depression Scale* (HADS [43]) The HADS is 14-item self-report measure of symptoms of anxiety and depression. Seven of its questions pertain to anxiety, whilst the other seven pertain to depression. Participants rate items on a 4-point Likert scale, from 0 (not at all) to 3 (most of the time). Examples of items are “I have lost interest in my appearance” and “I feel restless as if I have to be on the move.” Total scores for the anxiety and depression subscales can range from 0 to 21 on each subscale, with greater scores indicating greater anxiety or depression. Initially developed for patients with physical health problems, the HADS two-factor structure has been supported in ABI samples [56, 57]. The HADS has excellent internal consistency and strong convergent validity with other measures of depression and anxiety in TBI samples [58, 59].

The *Behavioural Inhibition Scale/Behavioural Activation Scale* (BIS/BAS [60];) is a 20-item measure that assesses behavioural approach or avoidance motivational systems that underlie behaviour. The BAS portion contains 3 subscales: drive (“I go out of my way to get things I want”), fun seeking (“I crave excitement and new sensations”) and reward responsiveness (“When I get something I want, I feel excited and energised”), whilst the BIS portion is a single subscale (“I worry about making mistakes”). Items are rated on 4-point Likert scale, ranging from 1 (strongly agree) to 4 (strongly disagree). Scores on the drive and fun seeking subscales range from 4 to 16, reward responsiveness from 5 to 20, with higher scores indicating greater activation. The BIS scale ranges from 7 to 28, with higher scores indicating greater inhibition. The BIS/BAS subscales have demonstrated good internal consistency, and the three BAS subscales load strongly on a second-order factor separate from the BIS [60]. The BIS/BAS also has adequate test-retest reliability and has demonstrated convergent and discriminant validity [60].

The *Intolerance of Uncertainty Scale—Short Form* (IU-SF [61];) is a 12-item measure of responses to uncertainty, ambiguous situations and the future. The IU-SF has two subscales: prospective IU (“I always want to know what the future has in store for me”) and inhibitory IU (“When it’s time to act, uncertainty paralyses me”). Items are rated on a 5-point Likert scale ranging from 1 (not at all characteristic of me) to 5 (entirely characteristic of me). Scores on prospective IU range from 7 to 35, and scores on inhibitory IU range from 5 to 25. Greater scores indicate greater difficulties with either prospective of inhibitory IU. The IU-SF has a stable two-factor structure and has demonstrated good internal consistency, convergent and discriminant validity [61, 62]. This measure was included to investigate whether changes in IU relates to changes in activity level (measured via the BADS) in ABI.

The *Impact of Events Scale-Revised* (IES-R [63]) is a 22-item measure that assesses post-traumatic symptoms within the past week. The IES-R has three subscales: intrusion (“Any reminders brought back feelings about [my injury]”), avoidance (“I tried not to talk about [my injury]”) and hyperarousal (“I was jumpy and easily startled”). Items are scored on a 5-point Likert scale ranging from 0 (not at all) to 4 (often). Scores on intrusion and hyperarousal range from 0 to 35, and scores on avoidance range from 0 to 40. The total scale ranges from 0 to 110, with greater scores indicating greater post-traumatic stress. Cronbach’s alpha of the subscales is high in patients with burn injuries [64] and inpatients with life-threatening cardiac events [65]. The IES-R has acceptable discriminative validity when distinguishing patients with and without PTSD after motor vehicle accidents (sensitivity = 0.74, specificity = 0.63) and has demonstrated good convergent, divergent and concurrent validity [66].

The *Brain Injury Rehabilitation Trust Motivation Questionnaire-Self* (BMQ-S [67]) is a 34-item questionnaire that measures levels of motivation in ABI populations. Questions are rated on a 4-point Likert scale, which participants can answer always, often, sometimes and never to questions. Total scores range from 34 to 136, with higher scores indicating greater difficulties with motivation (i.e. not motivated). Examples items include “I plan my week and make arrangements for things to do” and “Someone has to tell me what to do each day.” The BMQ-S has an internal consistency of 0.94 and has a strong relationship with the Apathy Evaluation Scale (r = 0.67). The BMQ-S has also been found to have good test-retest reliability (ICC = 0.85 [68];) and a high Guttman split-half reliability coefficient (0.90) [67].

The *Motivation for Traumatic Brain Injury Rehabilitation Questionnaire* (MOT-Q [69]) is a 31-item scale designed to measure motivation for rehabilitation activities in ABI. It consists of four subscales: lack of denial (“I have always had the problems I am having now” [reverse scored]), interest in rehabilitation (“Rehabilitation is very useful”), lack of anger (“Rehabilitation therapists can’t help me with my problems” [reverse scored]) and reliance on professional help (“I rely on doctors to help me with my problems”). It uses a 5-point Likert scale rated from − 2 to + 2 (strongly disagree to strongly agree). Total scores range from − 62 to + 62, with higher scores indicating greater motivation for rehabilitation. Chervinsky et al. [69] reported the MOT-Q total score had a Cronbach’s alpha of 0.91, and a Cronbach’s alpha of 0.86, 0.86, 0.83 and 0.73 for the subscales lack of denial, interest in rehabilitation, lack of anger and reliance on professional help, respectively. The MOT-Q has additionally been found to have good test-retest reliability [68].

The *Modified Outcome Measure—Participation Objective, Participation Subjective* (MOM-POPS [70];) is a shortened version of the original POPS scale. The POPS was originally designed to be a measure of community integration in individuals post-TBI, with the scale aimed at producing a Participation Objective score and a Participation Subjective score. In the MOM-POPS, participants are asked to rate their participation in household, occupational and social activities in the past week, based on (1) an estimate of the amount of household, occupational, and social activities engaged in within the past week (Participation Objective score);( 2) whether they would like to be doing more, less or the same of these activities (Participation Subjective score); and (3) whether these activities are the most, very, moderate, a little or not at all important to their satisfaction with life. Participants are then asked to circle from a list of options (e.g. cleaned the house, volunteer work, made social arrangements) which activities they have engaged in within the past week and have the option to list any additional activities they have engaged in. Although the difficulty of assessing the reliability and validity of a measure that provides both objective and subjective data has been discussed [70], the original POPS has shown acceptable internal consistency and good test-retest reliability and has good ecological validity.

The *Snaith-Hamilton Pleasure Scale* (SHAPS [71];) is a 14-item measure of hedonic capacity within the past few days. Items are rated either strongly disagree, disagree, agree or strongly agree. If a participant responds to either disagree category they receive a score of 1, and if they respond to either of the agree categories they receive a score of 0. Hence, the scale total range is 0 to 14, with higher scores representing greater anhedonia. Example items include “I would enjoy a warm bath or a refreshing shower” and “I would be able to enjoy a beautiful landscape or view.” The SHAPS has shown high internal consistency, convergent and discriminant validity and test-retest reliability in clinical and non-clinical populations [71–73].

The *Sense of Control Scale* (SCS [74, 75];) is a 12-item measure of an individual’s perceived ability to exert control over their life. It consists of two subscales: personal mastery (“I can do just about anything I really set my mind to”) and perceived constraints (“There are many things that interfere with what I want to do”). Items are rated on a 7-point Likert scale, ranging from strongly disagree to strongly agree. Total scores can range from 12 to 84. Factor analysis has supported its two-factor structure, with each subscale having adequate internal consistency (0.70 and 0.86, respectively [74]).

The *Credibility/Expectancy Questionnaire* (CEQ) is a 6-item measure of participant expectations of treatment outcome and perceived credibility of the treatment [76]. It consists of two factors, expectancy (“How much improvement in your symptoms do you really *feel* will occur?”) and credibility (“How logical does the therapy offered to you seem?”). The two-factor structure of the CEQ is supported, and has high internal consistency and test-retest reliability [76]. This measure will only be given at time 1.

The *Post-Study Questionnaire* (PSQ) was designed to obtain general feedback from the MAPLES trial. Participants will provide “top of mind” responses to questions focused around enjoyment of the group and factors involved in group attendance, such as “Do you see any benefits to being part of group sessions?” and “Do you feel there are any barriers to you participating in group sessions?” Participants are encouraged to provide any additional comments to support their answers, or comment on any topic that was not specifically addressed in the PSQ.

#### The exit interview

Approximately 20 participants will be randomly selected to take part in an exit interview at time 3. If a participant is selected to complete the exit interview, they will be asked a series of in-depth question to gain more specific feedback about the groups and their experiences within it. Examples and prompts are used to facilitate recall of the groups. The interview begins with general responses to group therapy, including items such as “Can you give your general thoughts on participating in the group?” and “Do you think [the therapist] had any impact on how you responded to the group?” If participants took part in the activity planning group, they were asked to comment specifically on the content and materials of the group, with questions around the value of mood monitoring, scheduling in activities, and barriers to homework completion. Finally, all participants are asked about practical aspects that may have affected their participation, such as a relationship with a partner or close friend, transport or health difficulties or their relationship with other group members.

Along with the above measures, participants in the activity planning group will be asked to submit a copy of their activity schedules that they will be completing as part of the group in order to provide a summary of types of activities engaged in as part of BA trial.

In order to promote participant retention, individuals who agree to take part will receive travel reimbursement for each session attended (time 1, 2, 3, and group sessions) as well as £50 compensation at the time 3 assessment. Scheduling of assessments and group sessions will be flexible based on the preference of trial participants’ availability; hence, the days in which group sessions occur will likely differ across phases of the trial. If a participant discontinues participation in the group sessions, the research team will approach them and ask if they are still interested in completing the outcome assessments (time 2 and time 3).

#### Data management

All participants will have a unique anonymised trial ID for the purposes of data collection and management. Data entry will be completed by an independent researcher with no knowledge of group allocation and will only be given the questionnaires labelled with the trial specific ID and be kept separately from any other trial documentation with a password not known to the researcher conducting the interventions. If any of the outcome measures have missing items, the total score will be estimated by using averaged responses from answered items. Any participant with more than 20% of items missing from any of the above measures (e.g. more than 2 items missing on a 10-item scale) at any time-point will be treated as a missing value.

Upon completion of data collection and entry, the dataset will be given to AK to match to the master list of participants in order to conduct analyses. Individuals that were first randomised to the waitlist control group and completed a new baseline will have their data restructured before analysis. Firstly, the time 2 assessment for waitlist control participants will still be used as is, but will also be used as a new time 1 assessment as part of the subsequent group (whether activity planning or activity engagement). Prior to analysis, all data will be double checked by AK against the raw questionnaire scores through double entry. Initial visualisation of the data and summary statistics will be used to detect extreme or impossible scores. Physical data (e.g. questionnaires) will be stored in a secure locked filing cabinet, separate to any personally identifiable information. All data will be stored on a secure drive within the department, accessible only to the research group,

#### Statistical methods

Quantitative and qualitative data analysis will be conducted by AK and supervised by senior members of the research team (TM, FCM, PVP) and the trial statistician (PW). No interim analyses are planned.

To determine the feasibility of the trial, percent estimates of recruitment rates will be calculated (number of participants consented vs numbers of eligible participants vs total potential participants). A CONSORT flow diagram will be used to visualise the number of participants screened, assessed for eligibility, found eligible, consented to participate, and subsequent allocation and assessment attendance. Percent attrition rates, what point attrition occurred and number of sessions attended per participant along with ratings of satisfaction within each group will be calculated to inform conclusions about the acceptability of the trial. Missing data points from the assessment period will also be reported. Protocol deviations and reasons for drop out and non-attendance (if available) will be reported.

Quantitative data will be analysed using R statistical software package [77]. Demographic variables of the participants allocated to each group will be reported. Means and standard deviations, both with 95% confidence intervals, of measures will be reported for all three groups at each time-point. Data will be analysed using an intention-to-treat analysis by including all randomised participants into our analysis. In order to determine if the treatment effect is constant at initial randomisation and at re-randomisation, average change scores will be reported for those first randomised to the waitlist control group and for those randomised directly to either the activity planning or activity engagement group.

An exploratory analysis will then be conducted to assess the effectiveness of the activity planning and activity engagement groups. A generalised linear mixed-effects model with maximum likelihood estimation and an unstructured covariance matrix using the primary outcome measure, the BADS, across the multiple assessment points will be used. As only those in the waitlist control group will be re-randomised, there are unlikely to be carry-over effects that influence treatment effects in the subsequent group allocation. Thus, treatment effects of these individuals are not expected to differ from participants allocated to the activity engagement and activity planning groups in the initial randomisation period. A residual covariance matrix will be used to investigate homogeneity of variance. Participant intercept and slope will be used as the random effects, with group status (activity planning vs activity engagement vs waitlist control) as the fixed effect. If the data is found to not have a linear structure and/or the random intercept, random slope model fails to converge, a random intercept model will instead be conducted. Although mixed-effects models provide appropriate type I error corrections, if the final sample size is smaller than anticipated, type 1 error rates will be corrected prior to conducting the analysis. Generalised eta squared will also be reported based on this analysis.

The minimal clinical important difference (MCID) of the BADS will additionally be estimated to inform a power analysis for a future definitive trial. The MCID will be used in conjunction with feasibility (i.e. recruitment rates) and acceptability (i.e. exit interviews) data in a decision on whether to progress to a main trial. Given the statistical limitations associated with using the MCID to inform a power analysis with small sample sizes [78], the MCID will not be estimated if 40 participants or less are recruited. All statistical results will be reported as purely exploratory and care will be taken to report all results as such.

The potential effect of demographic variables will also be explored. Change scores on the BADS will be summarised across gender, educational status, type of brain injury, injury severity and location of lesion/injury. The potential effect of time since injury on outcome measures will be summarised informally by whether individuals are within the first year, second year, third year or greater than 3 years-post injury. When exploring potential effects of the groups, change scores will be summarised between those with and without antidepressant medication in order to detect any potential influence of medication on scores. All subgroup results will be descriptive only, will be emphasised within the final paper as exploratory and will only be provided as a supplementary document.

Qualitative data from the exit interview will be used to inform the acceptability and practicality of the activity planning and activity engagement group. Data will be analysed using an interpretive description framework, a technique developed to identify clinically relevant information in complex health care populations [79]. The qualitative data will be organised into relevant themes that either facilitated or prevented a participant from benefitting from the trial.

Qualitative data will be approached with a constructivist epistemological stance. Interviews will be analysed using a constant comparative analysis and will follow the steps outlined by Braun and Clarke [80] in creating and coding themes. Any field notes or transcription notes will be used to supplement the analysis. In order to increase transferability, thick description of the participant demographics, interviewer experience and training and services provided at the recruitment sites will be reported. Given that approximately 30% of the sample (20 participants) will provide interviews, saturation will be determined when no new information meaningfully affects the thematic map. The building of themes will be triangulated using data from the quantitative outcome measures and interpretation will be formed using behavioural activation theory as an analysis framework. The interpretation of themes will be in part discussed with the Steering Committee service user in order to enhance the credibility and hence the trustworthiness of the results. Confirmability and dependability of the data will be supported through informal member checking with participants during the interview, and peer debriefing among the research team and clinicians working in ABI settings.

### Methods: monitoring

#### Data monitoring

A data management committee was found to be unnecessary, given the small scale of the trial, and as such will be managed by the Steering Committee. Given that the MAPLES trial is a pilot feasibility trial predominantly, a formal Data Monitoring Committee was deemed unnecessary given the modest number of participants expected to be recruited relative to the amount of data points. No interim analyses will be conducted. The trial lead, principal investigator and trial statistician (AK, TM, PW) will have full access to the final dataset. Results of the current trial will be reported as per CONSORT recommendations.

#### Harms

The management and reporting of adverse events will be conducted as per the recommendation of the UK Medical Research Council protocol. Although participants may experience distress discussing their difficulties with engaging in meaningful activities, this is considered a normal aspect of psychological therapies. Necessary precautions have been taken to reduce the chance of an adverse event occurring (i.e. excluding participants with suicidal intent). In the unlikely case of an adverse event, details will be reported in the final trial paper. Any concerns about the well-being of a participant will be discussed in both clinical supervisions and in Steering Committee meetings about the best way to ensure the best outcome for the participant.

#### Auditing

Auditing will be conducted as part of the Steering Committee meetings. Regular site visits (at least once every 2 months) will be completed in order to review referral and enrolment rates and consistent application of recruitment strategies. Auditing of safety procedures, including risk of harm, participant consent and safeguarding concerns of the trial will be investigated as part of the regular clinical supervisions of the intervention therapist (AK) and a clinical psychologist (TM), held weekly when groups are currently running.

## Ethics and dissemination

### Research ethics approval

This trial has received ethical approval from the Health Research Authority of the UK National Health Service (East of England—Cambridge Central, REC reference 18/EE/0305). The trial was registered at ClinicalTrials.gov on 12 March 2019 (NCT03874650) where the protocol can be accessed.

### Protocol amendments

Any amendments will be immediately reported to the trial sponsor, the recruitment sites and appropriately submitted to the Research Ethics Committee after approval from all Steering Committee Members has been given. Modifications to the protocol will additionally be reported on the protocol registration (NCT03874650).

### Consent

All participants recruited will be taken through an informed consent process prior to beginning participation in this trial and have the opportunity to withdraw consent at any time. Consent to participate will be obtained by a member of the research team.

### Confidentiality and access to data

The risk of a breach of confidentiality is managed by the research being conducted in accordance with best practice. Personally identifiable information (“PID”—names, addresses, dates of birth etc.) are kept strictly separate from fully anonymised research data (questionnaires, session recordings). PID are held in a secure “haven” on departmental university computer server and/or locked filing cabinets and will be retained for only 12 months after the last participant has completed the trial (in case of a need to re-contact), after which they will be deleted.

At the time of consent, we make potential participants aware that there are limits to our duty of confidentiality if information is disclosed that indicates a significant risk of harm to the participant or another individual. If such a disclosure occurs, we will follow our standard operating procedures of alerting the individual’s GP (in the case of self-harm) or appropriate protection agencies (in the case of harm to others). Paperwork and files relating to trial participants will be stored in locked filing cabinets at the university department, only accessible by the research team. Personally identifiable data and anonymised data will be stored separately in order to decrease the risk of breaches to confidentiality. Only the research team will have access to personal information (contact details) for the purposes of contacting participants. The research team will only access medical records once for the purposes of collecting injury-related information. Medical information will not be stored with participant contact information and will only be linked to anonymised participant indicators. The research data (questionnaires, administered in person, by post or online) will only contain anonymised participant indicators.

### Ancillary and post-trial care

In the unlikely event that a participant suffers harm from the trial, the trial sponsor will provide appropriate compensation. Local university and MRC policy will be followed to document and report the adverse event.

### Dissemination policy

All participants will be given the option to receive a summary of the trial results and have the opportunity to discuss them with the research team. Trial results will be shared in traditional methods such as journal publications, presentation at conferences and through social media. Results will also be disseminated to the recruitment sites, as well as to other research groups within the university. The final quantitative dataset along with the statistical code will be published and freely available to third parties for review. Qualitative transcripts will be made available only once transcripts have been sufficiently modified to remove any contextual information that might identify a participant (e.g. mention’s partner’s name, hometown, etc.).

### Limitations

Although many steps have been taken to reduce risk of bias, envelopes as a method of allocation concealment has been associated with bias in trials and thus should be considered when interpreting results.

## Discussion

Depression and low mood affect at least one third of individuals with an ABI (which may be a rather conservative estimate), and many of those with depression receive treatment [81, 82]. One of the major barriers to successfully treating depression and other mental health disorders in ABI is the need for an intervention that addresses the complex needs of the population [20]. Various studies have documented the link between lowered activity level and depression in ABI [28, 35, 83–85]; hence, investigating whether BA is a feasible, acceptable and potentially efficacious treatment is warranted—particularly with group delivery, as is done here. The simplicity, whether combined with strategies to enhance planning and engagement in meaningful activities or not, and the fact that it does not need to be delivered by a clinical psychologist [86], make it a potentially attractive option for service provision that has the potential to immediately influence and benefit ABI services.

Understanding differences in feasibility and research outcomes between the activity planning and activity engagement groups is also essential to the development of affordable and effective treatments. The activity engagement group does not explicitly highlight the relationship between low mood and activity level; hence, it may produce short-term mood gains that do not generalise to changes in everyday life—the ultimate aim of BA. However, in the context of low levels of activity that frequently accompany ABI, it is possible that simply experiencing positive reinforcement through social engagement will lead to explicit or implicit learning of the relationship between mood and activity level that could foster increased activity engagement beyond the group. It is notable that implicit forms of learning are often relatively well preserved even when explicit memory is highly compromised [87]. The cognitive demands of the activity planning group, even when modified for ABI, may negatively affect acceptability relative to the activity engagement group. If the activity engagement group is well accepted, recreating and running such a group could be easily recreated without extensive preparation and cost.

Providing initial data on *why* a group intervention is well accepted is also important. Within a standard single group vs treatment-as-usual or waitlist control design, the question would arise of whether it was the BA component or simply the social experiences of being in a group that was responsible for any gains. The current trial design, by contrast, would allow exploration of factors associated with group attendance, attrition and potential variables of change between groups. Similarly, if the groups are both comparably well accepted, we might conclude that the activity scheduling elements etc. of BA may add little beyond the social group activity context in terms of feasibility. Were the activity engagement group to prove most effective, we would have stronger grounds to conclude that the cognitive elements (i.e. planning and organisational skills) used in BA and the more business-focused nature of the sessions may have undermined its acceptability in ABI.

## Trial status

The first participant consented to the MAPLES trial on May 20, 2019. Recruitment is planned to continue until April 2021.

## Supplementary information

**Additional file 1.** MAPLES Pilot Feasibility Trial Fidelity Assessment Checklist. Activity Planning Group.

**Additional file 2.** MAPLES Pilot Feasibility Trial Fidelity Assessment Checklist. Activity Engagement Group.

## Data Availability

Not applicable to the protocol. The dataset generated will be made available to the public upon completion of the trial on the trial’s protocol registration record (NCT03874650).

## Footnotes

1 This includes traumatic brain injury, stroke, anoxia/hypoxia, encephalitis and other brain infections, and chemotherapy-related neurotoxicity and resected brain tumours.
