# Supplementary material for: Mood, Activity Participation, and Leisure Engagement Satisfaction (MAPLES): a randomised controlled pilot feasibility trial for low mood in acquired brain injury
Source: Pilot Feasibility Stud. 2020 Sep 22;6:135. doi: 10.1186/s40814-020-00660-8 (PMC7507282; doi:10.1186/s40814-020-00660-8)
Supplement: Supplementary file 1 — Additional file 1. MAPLES Pilot Feasibility Trial Fidelity Assessment Checklist. Activity Planning Group. [file 40814_2020_660_MOESM1_ESM.docx]

**MAPLES Pilot Feasibility Trial Fidelity Assessment Checklist**

**Activity Planning Group**

Rating Instructions: Upon listening to each audiorecroding for each session, rate each aspect on whether they occurred as below:

0: Component not present/not attempted

1: Therapist attempted component partially/questionably

2: Therapist covered component and relevant content

A new checklist should be completed for each cohort of the Activity Planning Group. Scores are to be compared across and within cohorts of the group.

**Session 1: Introduction to Group Therapy**

**Cohort Number:**

**Date:**

____ Introduction to the group structure

____ Introduction of group members

____ Introduce link between activity level and mood

____ Discussed examples as relevant to participants

____ Rationale of behavioural activation

____ Education on executive function difficulties in brain injury

____ Introduce concept of absentmindedness

____ Introduce concept of mood and mind monitoring

____ Explained and set homework tasks

**Session 2: Identifying Enjoyable Activities**

**Cohort Number:**

**Date:**

____ Reviewed mood and mind monitoring homework from last week

____ Discuss relationship between mood and attention for each group member

____ Introduce value-based activity planning

____ Discussed examples as relevant to participants

____ Introduce Activity Wheel to identify core values

____ Introduce link between core values and personal goals

____ Participants identify short-term goals within the group and long-term goals

____ Participants begin activity scheduling and learn to break down activity into steps

____ Explained and set homework tasks

**Session 3: Changing Habits and Planning Pleasurable Activities**

**Cohort Number:**

**Date:**

____ Reviewed activity scheduling homework from last week

____ Provide example of how absentminded errors occur in daily life

____ Introduce concept of the automatic pilot

____ Discussed examples as relevant to participants

____ Introduce identifying personal triggers to absentminded errors

____ Participants identify personal triggers to absentminded errors within group

____ Participants schedule another activity and break down activity into steps

____ Explained and set homework tasks

**Session 4: Goal Review and Balancing Enjoyable and Routine Activities**

**Cohort Number:**

**Date:**

____ Reviewed activity scheduling homework from last week

____ Review homework on identifying personal triggers for absentminded errors

____ Education on preventing personal triggers

____ Discussed examples as relevant to participants

____ Participants create personalised strategies to prevent triggers

____ Participants review progress on goals from session 2

____ Group discussion on whether activities in day-to-day life align with values/goals

____ Introduce concept of balancing between routine and enjoyable activities

____ Participants schedule another activity and break down activity into steps

____ Explained and set homework tasks

**Session 5: Identifying Solutions to Goal Attainment**

**Cohort Number:**

**Date:**

____ Reviewed activity scheduling homework from last week

____ Review homework on preventing personal triggers

____ Education on falling into avoidance patterns

____ Discussed examples as relevant to participants

____ Participants create personalised strategies to reverse avoidance patterns

____ Participants review concept of the automatic pilot

____ Participants learn STOP acronym to prevent distraction from goal attainment

____ Participants practice using STOP acronym

____ Participants schedule another activity and break down activity into steps

____ Explained and set homework tasks

**Session 6: Increasing Mastery and Managing Fatigue**

**Cohort Number:**

**Date:**

____ Reviewed activity scheduling homework from last week

____ Review homework on reversing avoidance patterns

____ Education on importance of improving mastery and risks of plateauing

____ Discussed examples as relevant to participants

____ Participants identify “warning signs” of plateauing

____ Education on different types of fatigue post-brain injury

____ Participants identify personalised triggers to fatigue

____ Participants develop personalised strategies to manage fatigue

____ Participants schedule another activity and break down activity into steps

____ Explained and set homework tasks

**Session 7: Active Approaches to Engagement**

**Cohort Number:**

**Date:**

____ Reviewed activity scheduling homework from last week

____ Review homework on attempting strategies to prevent fatigue

____ Education on importance of social relationships in maintaining a good mood

____ Discuss examples as relevant to participants

____ Education on active versus passive approaches to activity scheduling

____ Discussion on personal barriers to taking an active approach

____ Participants practice in session how to initiate a social activity

____ Participants list benefits of the active approach and drawbacks of passive approach

____ Participants schedule another activity and break down activity into steps

____ Explained and set homework tasks

**Session 8: Relapse Prevention**

**Cohort Number:**

**Date:**

____ Reviewed activity scheduling homework from last week

____ Review homework on active and passive approaches

____ Participants identify “take home” message from group

____ Review behavioural activation model from Session 1

____ Review value-based activity planning from Session 2

____ Review concept of automatic pilot from Session 3

____ Review concept of personalised triggers from Session 4

____ Review STOP acronym from Session 5

____ Review importance of increasing mastery from Session 6

____ Review importance of taking an active approach to activities from Session 7

____ Review importance of breaking down activity into manageable steps

____ Education on importance of preventing relapse into lower activity levels

____ Participants identify personal triggers that may cause a relapse

____ Participants create personalised strategies to prevent relapse triggers

____ Participants schedule another activity and break down activity into steps
