# Supplementary material for: Mood, Activity Participation, and Leisure Engagement Satisfaction (MAPLES): a randomised controlled pilot feasibility trial for low mood in acquired brain injury
Source: Pilot Feasibility Stud. 2020 Sep 22;6:135. doi: 10.1186/s40814-020-00660-8 (PMC7507282; doi:10.1186/s40814-020-00660-8)
Supplement: Supplementary file 2 — Additional file 2. MAPLES Pilot Feasibility Trial Fidelity Assessment Checklist. Activity Engagement Group. [file 40814_2020_660_MOESM2_ESM.docx]

**MAPLES Pilot Feasibility Trial Fidelity Assessment Checklist**

**Activity Engagement Group**

Rating Instructions: Upon listening to each audiorecroding for each session, rate each aspect on whether they occurred as below.

The Activity Engagement Group is flexible by nature, and this should include:

- No mention behavioural activation/activity scheduling
- No encouragement of direct planning beyond the group
- Therapist encouragement of engagement on activities within the group
- Therapist encouragement of participant autonomy of activities chosen
- Non-linear structure of groups

For the first two components, rate as below:

2: Component was not present

1: Therapist attempted component partially/questionably

0: Therapist covered component adequately

For the last three components, rate as below:

0: Component was not present

1: Therapist attempted component partially/questionably

2: Therapist covered component adequately

A new checklist should be completed for each session of each cohort of the Activity Engagement Group. Scores are to be compared across and within cohorts of the group.

**Cohort Number:**

**Session Number:**

**Date:**

____ No mention of behavioural activation/activity scheduling

____ No encouragement of direct planning beyond the group

____ Therapist encouragement of engagement on activities within the group

____ Therapist encouragement of participant autonomy of activities chosen

____ Non-linear structure
